# Research on Abnormal State Detection of CZ Silicon Single Crystal Based on Multimodal Fusion

**DOI:** 10.3390/s24216819

**Published:** 2024-10-23

**Authors:** Lei Jiang, Haotan Wei, Ding Liu

**Affiliations:** 1 School of Automation and Information Engineering, Xi’an University of Technology, Xi’an 710048, China; 2220321203@stu.xaut.edu.cn (H.W.); liud@xaut.edu.cn (D.L.); 2 Crystal Growth Equipment and System Integration National & Local Joint Engineering Research Center, Xi’an University of Technology, Xi’an 710048, China

**Keywords:** Czochralski silicon single crystal, multimodal fusion, wavelet transform, deep learning

## Abstract

The Czochralski method is the primary technique for single-crystal silicon production. However, anomalous states such as crystal loss, twisting, swinging, and squareness frequently occur during crystal growth, adversely affecting product quality and production efficiency. To address this challenge, we propose an enhanced multimodal fusion classification model for detecting and categorizing these four anomalous states. Our model initially transforms one-dimensional signals (diameter, temperature, and pulling speed) into time–frequency domain images via continuous wavelet transform. These images are then processed using a Dense-ECA-SwinTransformer network for feature extraction. Concurrently, meniscus images and inter-frame difference images are obtained from the growth system’s meniscus video feed. These visual inputs are fused at the channel level and subsequently processed through a ConvNeXt network for feature extraction. Finally, the time–frequency domain features are combined with the meniscus image features and fed into fully connected layers for multi-class classification. The experimental results show that the method can effectively detect various abnormal states, help the staff to make a more accurate judgment, and formulate a personalized treatment plan for the abnormal state, which can improve the production efficiency, save production resources, and protect the extraction equipment.

## 1. Introduction

**Figure 1:**
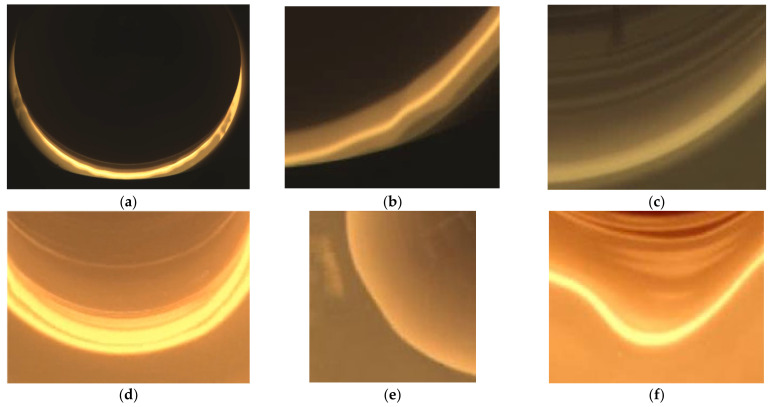
Solid–liquid interface diagram in the equal-diameter stage: (**a**) the crystal-pulling screen in the equal-diameter stage; (**b**) a normal growth picture of silicon single crystal; (**c**) a picture of silicon single-crystal loss; (**d**) a picture of silicon single crystal swinging during growth; (**e**) a picture of silicon single-crystal squareness during growth; (**f**) a picture of silicon single-crystal twisting during growth.

Silicon single crystals are indispensable semiconductor materials in new energy sources, storage, and electronics [1]. The Czochralski (CZ) method is the primary technique for preparing large-size silicon single crystals [2], which involves melting polycrystalline silicon in a quartz crucible under complex conditions, including high temperatures, vacuums, magnetic fields, and inert gases. A seed crystal is then immersed in the solution and slowly pulled upward at a specific speed, allowing silicon atoms to arrange along the seed crystal’s direction, forming a single crystal with a specific crystal orientation [3]. The growth process of silicon single crystals using the CZ method comprises six main stages: seeding, necking, shoulder release, shoulder rotation, equal diameter, and tailing [4]. Among these, the equal diameter stage occupies the majority of the crystal-pulling process time. Figure 1a illustrates the crystal-pulling picture in the equal-diameter stage. During the growth of silicon single crystals with a <100> crystal orientation, four crystal lines with 90° intervals between each other are generated on the crystal rod [5]. The solid–liquid interface diagram in the equal-diameter stage is depicted in Figure 1. The ability to effectively and timely detect and classify abnormal conditions during the growth of CZ silicon single crystals is crucial for improving crystal-pulling efficiency and protecting crystal-pulling equipment. In the current production process, the identification of abnormal conditions primarily relies on operators’ experience. This dependence leads to low accuracy in judging the crystal pulling state and delayed discovery of abnormal conditions, which negatively impacts crystal pulling efficiency and potentially damages the equipment.

In the equal-diameter stage of producing silicon single crystals using the Czochralski method, due to inappropriate process parameters and external disturbances, a series of abnormal conditions that affect crystal quality and production efficiency will occur, among which loss, twisting, swinging, and squareness are four common abnormal conditions. Loss is usually caused by impurities, temperature abnormalities, pulling speed fluctuations, etc.; swinging is usually caused by external disturbances and other factors; squareness is mainly caused by high pulling speed; and twisting is usually caused by low temperature, high pulling speed, mechanical failure, etc. [6]. For the two abnormal conditions of loss and twisting, the quality of the silicon single crystal is changed, and crystal pulling should be stopped and remelting measures should be taken; for the two abnormal conditions of swinging and squareness, when the degree is relatively mild, process parameters can be used to compensate, and when the degree is serious, crystal pulling needs to be stopped and remelting measures should be taken.

Several researchers have focused on loss detection to ensure the normal growth state of silicon single crystals. Jun Zhang et al. proposed a deep learning-based detection method to detect loss problems during the growth of solar-grade silicon single crystals, achieving an accuracy of 97.33% [7]. S Yuting et al. introduced an improved Yolov4-Tiny model (Yolo-SPI) to detect loss occurrences, with a detection accuracy of 98.01% [8]. However, both of these networks only utilize the single-dimensional video signal for loss detection. Lei Jiang et al. proposed a multimodal fusion method for offline detection during silicon single-crystal growth. By using the fusion decision of image signals, temperature signals, pulling speed signals, and diameter signals, they detected loss phenomena with an accuracy of 98.36 [9].

Although the above researchers have achieved excellent results in the loss detection of silicon single crystals, other common abnormalities in the growth process of silicon single crystals, such as swinging, twisting, and squareness, can also affect the quality of the crystals. Currently, few researchers have addressed the classification of these three common abnormal conditions. Moreover, the existing detection methods often rely on single-dimensional signals, which may not capture the full complexity of the crystal growth process. The use of multiple data sources and their fusion could potentially provide more comprehensive and accurate detection and classification of abnormal conditions.

The method for classifying one-dimensional signals can be selected according to the characteristics of the signal and the application scenario. In the time domain, classification can be performed based on the mean, standard deviation, maximum value, minimum value, zero crossing rate, etc. [10]. In the frequency domain, classification can be performed by extracting frequency domain features through Fourier transform. In the time–frequency domain, wavelet transform is used to extract features at different scales for classification. In terms of traditional machine learning algorithms, one-dimensional signals can be classified using methods such as support vector machine (SVM), decision tree, and random forest [11]. For the classification method of image signals, the convolutional neural network is currently the core algorithm model for image classification. At present, various improved convolutional neural networks have been proposed, such as ResNet [12], Swin Transformer [13], and ConvNeXt [14], which have performed well in various image classifications and have better classification accuracy. For resource-constrained environments, researchers have developed lightweight networks such as DenseNet [15], MobileNet [16], ShuffleNet [17], and EfficientNet [18]. Multimodal fusion utilizes the information complementarity of each modality to achieve more comprehensive and accurate information processing and decision support. The fusion of different modal information can be divided into data layer fusion, feature layer fusion, and decision layer fusion according to the fusion stage. In data layer fusion, the original data of multiple modalities are directly fused, and in feature layer fusion, the original data are fused after certain feature extraction operations [19]. In feature layer fusion, multiple layers of the features of a single modality can be fused to strengthen feature information, or the attention mechanism can be used to focus on important channel features in a certain layer of features [20,21,22]. In decision layer fusion, multiple decision results are obtained after the feature extraction of multiple modalities, and the final decision result is obtained by fusing multiple decision results [23].

To address the limitations in existing studies of silicon single crystals’ abnormal growth state classifications, we propose a multimodal fusion classification network that leverages multiple data sources, including one-dimensional signals (diameter, temperature, and pulling speed) and video data (meniscus images and frame difference images). First, we employ continuous wavelet transform to extract time–frequency domain features of one-dimensional signal diameter signal, temperature signal, and pulling speed signal. A novel convolutional neural network Dense-ECA-SwinTransformer is proposed to process and extract time–frequency images and output one-dimensional signal abnormal features. Second, to extract abnormal features from meniscus video signals, the ConvNeXt networks is employed in the paper to process meniscus image signals and inter-frame difference image signals and output video abnormal features. Finally, we designed a novel multimodal fusion network to fuse the extracted one-dimensional signal abnormal features and video abnormal features. Leveraging attention mechanisms, the proposed multimodal fusion network can fully consider abnormal signals across different modalities and realize abnormal state category detection and classification.

To validate our proposed methods, we conducted extensive experiments using a dataset collected from real silicon single-crystal growth processes. The experimental results demonstrate that our multimodal fusion approach significantly outperforms single-modality methods, achieving an overall classification accuracy of 99.2% for abnormal growth states. The Dense-ECA-SwinTransformer fusion model shows a 2.1% improvement in accuracy compared with only using one-dimensional signals. Furthermore, our fusion approach for video signal processing yields a 1.8% increase in detection accuracy for subtle abnormalities compared with using meniscus images alone.

The main contributions of this article are as follows:

- Advanced Multimodal Fusion Network: We propose a novel multimodal fusion classification network for fault phenomenon detection in CZ silicon single-crystal growth. This network integrates time–frequency domain feature maps from one-dimensional signals with image and inter-frame difference signals from video data, implementing a channel attention mechanism for enhanced abnormal state detection and classification;
- Innovative Signal Processing Techniques: We introduce advanced processing techniques for both one-dimensional and video signals. This includes applying continuous wavelet transform to one-dimensional signals and applying transformers to the classification of abnormal states of silicon single-crystal growth for the first time, converting video signals into image signals and inter-frame difference signals for more nuanced abnormal state detection;
- According to the results of this study, we detect and classify abnormal states by fusing multimodal data. Compared with only using one-dimensional signals or video signals, the detection and classification model proposed in this paper has better robustness and accuracy.

## 2. Data Collection

**Figure 2:**
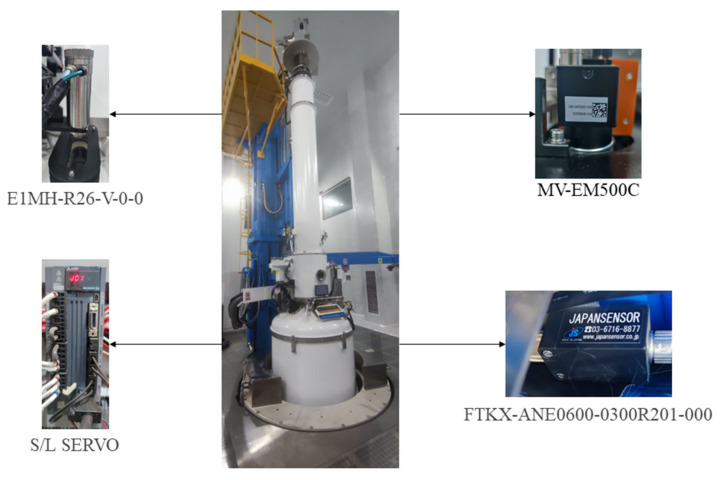
CZ single-crystal furnace equipment and corresponding sensors: high-temperature infrared sensor E1MH-R26-V-0-0 collects diameter data, S/L SERVO collects speed data, FTKX-ANE0600-0300R201-000 collects temperature data, and camera MV-EM500 collects meniscus data.

The experimental data come from the production of 12-inch series single-crystal furnace. The diameter data are collected using the high-temperature infrared sensor E1MH-R26-V-0-0 which purchased from Fluke in the Everett, WA, USA, the pulling speed data are obtained through S/L SERVO which purchased from Mitsubishi Group in Tokyo, Japan, the temperature data are collected using the term temperature sensor model FTKX-ANE0600-0300R201-000 which purchased from JAPANSENSOR CORPORATION in Konan, Japan, and the meniscus signal is collected using the camera model MV-EM500 which purchased from Microvision in Beijing, China, as shown in Figure 2.

The equipment can produce 100–308 mm CZ silicon single crystals. The diameter detection range is 4–350 mm. The diameter of the quartz crucible is 800 mm and the maximum charge of poly silicon is 450 kg. The maximum power of the side heater of the equipment is 180 kw. The maximum power of the bottom heater of the equipment is 80 kw. The crucible speed adjustment range is 0–15 rpm, the crystal speed adjustment range is 0–20 rpm, the crucible lifting speed adjustment range is 0–1.3 mm/min, the crystal lifting speed adjustment range is 0–6 mm/min, the ultimate vacuum degree is 0.3 Pa, the air intake adjustment range is 0–200 L/min, the maximum magnetic field strength is 4000 Gauss, the crystallizer lifting stroke is 2.8 m, and the crucible lifting stroke is 750 mm. The temperature adjustment is determined according to the input amount of silicon material, and the adjustment range is usually 800–2500 (dimensionless).

**Figure 3:**
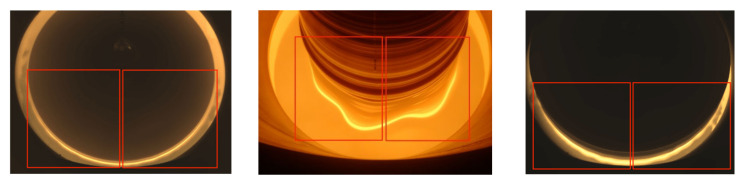
Pictures of the equal diameter stage, the images in the red box are used as the input image.

The experimental data used in this study are the one-dimensional signal data collected during the growth of the CZ silicon single crystal and the image of the meniscus video signal data surface. Figure 3 shows the image of the normal growth in the diameter stage of the crystal-pulling process. It can be seen from the figure that only the lower half can show the solid–liquid interface image, and with the growth of the crystal rod or the different camera angles, part of the solid–liquid interface in the middle will be blocked. Based on the above situation, the images in the red box in Figure 3 are used as the input image.

According to the one-dimensional signal dataset collected by the sensor, including diameter, temperature, and pulling speed, the corresponding number of one-dimensional related signals are collected according to the corresponding image set signals. Since the CZ silicon single-crystal growth system is a large time-delay system, for the diameter signal, the data point used corresponds to the image signal collection time point; for the temperature signal, it is 40 min forward of the current image signal collection point; for the pulling speed signal, it is 8 min forward of the current image collection point. The time alignment of the one-dimensional signal data and the image signal data is guaranteed.

This experimental environment uses the operating system Windows 10(21H1), the CPU is Inter(R) Core(TM) i7-10700 which purchased from Intel in the United States, the memory is 16G, and it runs on a GeForce RTX 3060 GPU workstation which purchased from NVIDIA in the United States. The CUDA version used is 11.3, the deep learning framework used is Pytorch1.9, and the data preprocessing tool and network building tool used are Python, version 3.7. During the training process of this network, the dataset is divided into a ratio of 7:2:1 for the training set: validation set: and test set, the batch size is set to 16, and the Adam optimizer is used. The optimizer has a learning rate of 0.0001, and the maximum training round is set to 100 times. If the loss does not decrease within five consecutive epochs, the training is stopped.

## 3. Multimodal Fusion Classification Model

**Figure 4:**
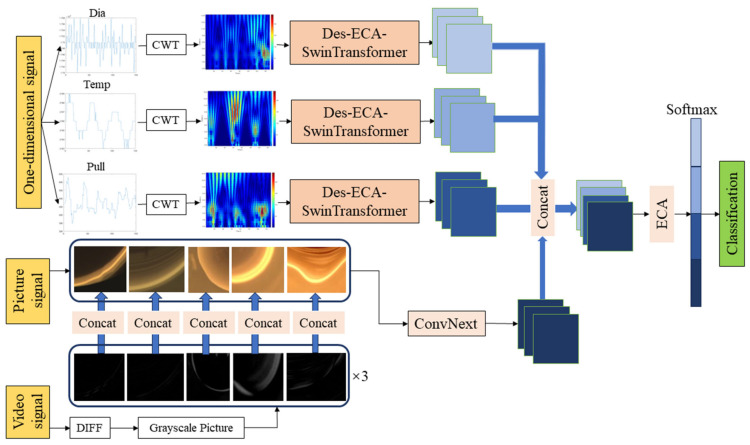
Overall framework of a multimodal fusion classification model.

To effectively detect and classify the state of the CZ silicon single-crystal growth process, this paper proposes a multimodal fusion multi-classification network, and its overall framework is shown in Figure 4. During the growth of CZ silicon single crystals, the time–frequency domain graph is obtained by continuous wavelet transform of the collected one-dimensional diameter signal, pulling speed signal, and temperature signal. This paper proposes a Dense-ECA-SwinTransformer neural network module to extract features from the time–frequency domain graph. It combines dense blocks and SwinTransformer modules and adds an ECA attention mechanism. The meniscus video signal collected by the camera is processed into an image signal and an inter-frame difference signal. First, the video signal is fused with the inter-frame difference image at the same time at the channel level and sent to the ConvNeXt neural network for feature extraction. After feature extraction, concatenation is used to fuse the feature graph extracted from the one-dimensional signal time–frequency domain graph with the feature graph extracted from the meniscus image. The ECA attention mechanism is added to the fused feature graph, so that the model pays more attention to providing more effective channels, further improving the model's ability to distinguish. Finally, it is sent to Softmax to obtain the state category of the CZ silicon single-crystal growth process.

### 3.1. Continuous Wavelet Transform

The continuous wavelet transform is essentially a convolution of a one-dimensional signal with a translation-shrinkage wavelet basis function that has localized properties in the time domain and frequency domain, thereby providing local information of the signal in both the time and frequency domains. Mathematically, the continuous wavelet function is defined as

(1) $$CWT(a,b)=\int_{-\infty }^{\infty }f(t)\psi_{a,b}^{*}(t)dt$$

Among them, f(t) is the input signal, $\psi_{a,b}(t)$ is the wavelet function generated by the mother wavelet through the scaling parameter a and the translation parameter b, $\psi_{a,b}^{*}(t)$ represents the complex conjugate of the wavelet function, parameter a controls the scale (frequency) of the wavelet, and parameter b controls the position (time) of the wavelet. The mother wavelet is a zero-mean, localized function. In this paper, the data length is 150 data points, and the Morlet wavelet is used. The wavelet scale ranges from 2 to 14, with every 0.5 as an interval. The 1250 × 938 × 3 time–frequency domain graph is generated by continuous wavelet change of one-dimensional data.

### 3.2. Dense-ECA-SwinTransformer

**Figure 5:**
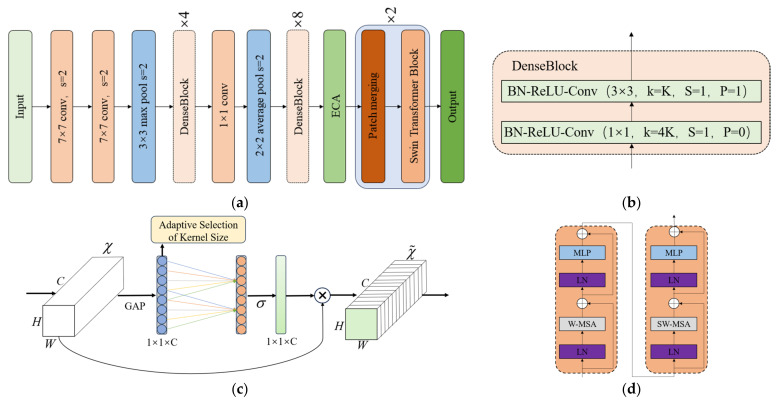
The structure of the network module: (**a**) Dense-ECA-SwinTransformer network structure; (**b**) Dense Block; (**c**) the ECA attention mechanism; (**d**) SwinTransformer Block.

The 1250 × 938 × 3 time–frequency domain image is resized to a 224 × 224 × 3 image, which is then sent to Dense-ECA-SwinTransformer for feature extraction. The structure of this network module is shown in Figure 5a, which involves three modules: DenseBlock, ECA attention mechanism, and SwinTransformer Block.

To extract the features of the one-dimensional signal time–frequency domain graph, we first pass through a 7 × 7 convolution layer with a stride of 2 and a 3 × 3 maximum pooling layer with a stride of 2. Then, we use dense blocks for feature extraction. Dense blocks establish direct connections between layers so that the features of the previous layer can be reused by subsequent layers, thereby reducing feature redundancy and improving the efficiency of the model. The network structure of the dense block is shown in Figure 5b. First, there is a convolution layer with a convolution kernel size of 1 × 1, the number of convolution kernels is 4K, K is the growth rate, which is used to control the number of feature channels output by the dense block, the stride is 1, the padding is 1, and then a 3 × 3 convolution layer with K convolution kernels, a stride of 1, and a padding of 0 is used. For each convolution layer, normalization is performed first, then nonlinearization, and finally convolution operation is performed. In this paper, two dense connection layers are used, one dense connection layer consists of 4 dense connection blocks, and the other dense connection layer consists of 8 dense connection blocks.

After using dense blocks for feature extraction, the ECA attention mechanism is introduced. This attention mechanism uses one-dimensional convolution to perform local cross-channel interactions. By adaptively selecting the size of the convolution kernel to capture important inter-channel dependencies, the ECA mechanism can better capture and focus on important local features and improve the quality of feature expression. The ECA attention mechanism is shown in Figure 5c. In the ECA module, the input is represented by $\chi \in {\mathbb{R}}^{H\times W\times C}$, H×W represents the size of the feature map, C represents the number of channels, σ represents the Sigmoid function, and the output is defined by $\tilde{\chi }$. The specific operation is as follows: global average pooling is performed on the input features to generate a global feature description for each channel. One-dimensional convolution uses 1D convolution with adaptively selected convolution kernel size to interact between channels, calculate the attention weight, and apply the calculated weight to the original feature to obtain the weighted output.

After using the ECA attention mechanism to assign channel weights, two Patch Merging and SwinTransformer modules are used for feature extraction. The role of the Patch Merging layer is to downsample, reduce the resolution, adjust the number of channels, and form a hierarchical design. The SwinTransformer Block uses a shifted window-based self-attention mechanism to divide the input feature map into non-overlapping windows and calculate self-attention within each window. The shift operation in the alternating layer realizes cross-window connections, enhancing the model's ability to capture long-distance dependencies while maintaining computational efficiency. The module is shown in Figure 5d. Each SwinTransformer block consists of a window multi-head attention mechanism (W-MSA), a shifted window multi-head attention mechanism (SW-MSA), layer normalization (LN), and a multi-layer perceptron (MLP), and is connected through residuals.

### 3.3. Image Signal Feature Extraction Module

**Figure 6:**
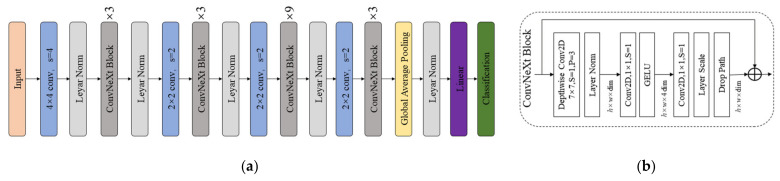
(**a**) ConvNeXt network structure diagram; (**b**) ConvNeXt Block.

This paper uses the ConvneXt neural network module to classify image information. The ConvNeXt network structure diagram is shown in Figure 6a.

The ConvNeXt Block is proposed in this network, as shown on the right of Figure 6. In the ConvNeXt Block, an inverse bottleneck structure and a residual structure are used. First, a DW convolution of size 7 × 7 and stride 1 is used, and then the LN layer is used for normalization. The second layer consists of a convolution kernel of size 1 × 1 and stride 1 for convolution, and the GELU function is used for activation. At the same time, the number of channels is increased to 4 times the original. The third layer uses a convolution operation of size 1 × 1 and stride 1 to reduce the dimension to the initial number of channels of the ConvNeXt block, forming an inverse bottleneck structure. Then, a Layer Scale layer is added to achieve the scaling of data in each channel through learnable parameters, and finally, the Drop Path is used for regularization.

## 4. Discussion

The experimental data used in this study are one-dimensional signal data collected during the growth of a single silicon crystal and images of the meniscus video signal data surface. After processing the collected image data, a total of 2814 groups of normal growth images, 2723 groups of loss images, 2474 groups of swinging images, 2566 groups of twisting images, and 1942 groups of squareness images were obtained. Each group of images included a meniscus image, an inter-frame difference image, a diameter time–frequency domain image, a temperature time–frequency domain image, and a pulling speed time–frequency domain image. To verify the effectiveness of the model and the accuracy of the detection and classification, we used the most widely used evaluation indicators, including accuracy, recall, precision, F1Score, and confusion matrix.

### 4.1. State Multi-Classification of One-Dimensional Signal

**Figure 7:**
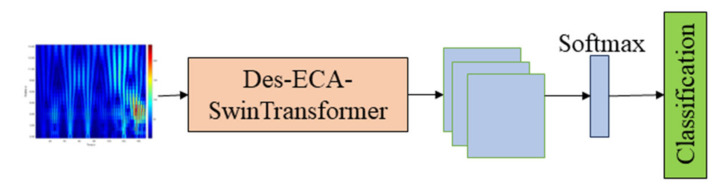
One-dimensional signal classification network structure diagram.

The diameter signal, temperature signal, and pulling speed signal were used separately for multi-state classification, and the Dense-ECA-SwinTransformer neural network proposed by us was used for classification. The classification network is shown in Figure 7.

**Figure 8:**
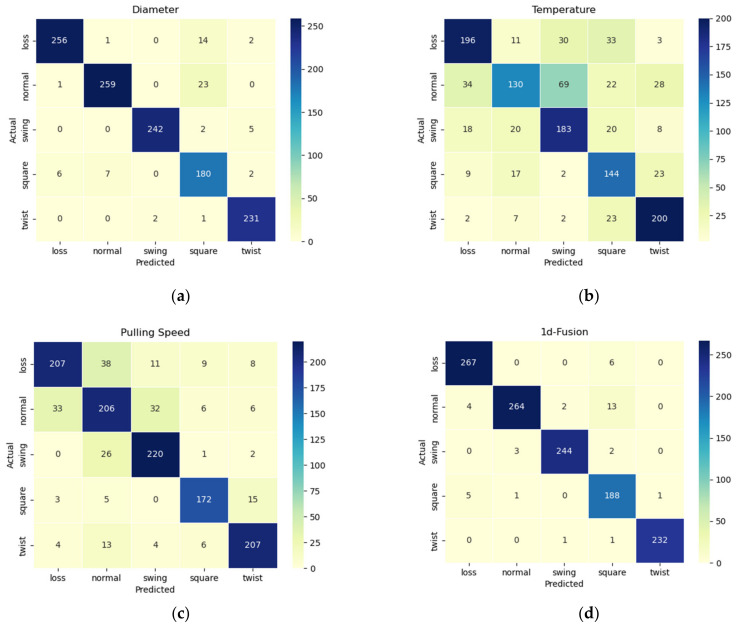
Confusion matrix of one-dimensional signal classification: (**a**) diameter; (**b**) temperature; (**c**) pulling speed; (**d**) one-dimensional signal fusion.

The classification results are shown in Table 1 below, and the confusion matrix is shown in Figure 8.

It can be seen from the classification results that the diameter signal has the most accurate classification result for the abnormal state, the pulling speed signal has a poor classification result for the abnormal state, and the temperature signal has the worst classification result for the abnormal state.

**Figure 9:**
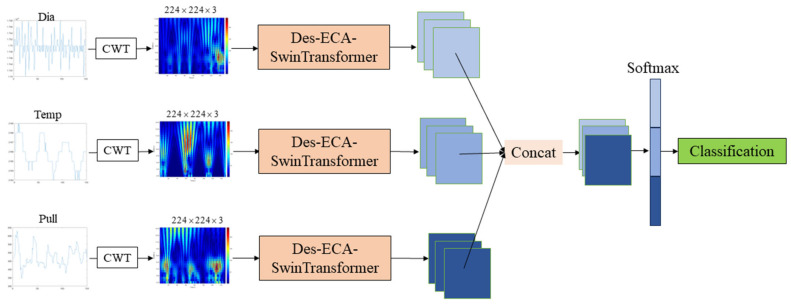
One-dimensional signal fusion network structure diagram.

From the confusion matrix of temperature signal classification, it can be seen that the classification effect of temperature signals for normal, swinging and loss is poor, but the classification effect for squareness and twisting is relatively good. From the confusion matrix of pulling speed classification, it can be seen that the pulling speed signal has a good classification effect for squareness, swinging, and twisting, but the detection of loss is poorly distinguished from the normal state. From the confusion matrix of the diameter signal, it can be seen that the classification effect of the diameter signal for each state is best among the one-dimensional signals. In summary, using only one-dimensional diameter signals, temperature signals, and pulling speed signals has different defects in the classification of abnormal states, which affects the classification accuracy. Therefore, the one-dimensional signal is extracted and fused to make the decision. The network structure diagram is shown in Figure 9.

The classification accuracy of a one-dimensional signal with a single dimension is the highest at 94.7%, and the classification accuracy is 96.8% after the processing and fusion of the one-dimensional signal. However, the confusion matrix of the one-dimensional signal confusion classification in the lower right corner of Figure 8 shows that although the classification effect is improved after fusion, there are still misclassifications in the classification of loss, normal, swinging, and squareness.

### 4.2. Multi-State Classification of Meniscus Images

**Figure 10:**
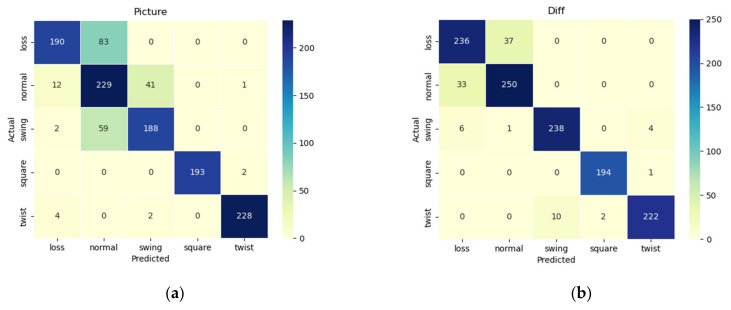

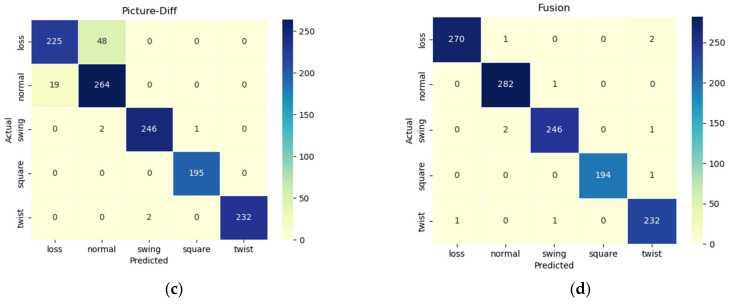
Confusion matrix: (**a**): meniscus image; (**b**): difference image; (**c**): fusion of meniscus image and difference image; (**d**): fusion of all data.

To classify the state of the silicon single-crystal growth process, in addition to using a one-dimensional diameter signal for classification, image signals and video signals are also important parts of the state detection of the CZ silicon single-crystal growth process. This study used the meniscus image signal of a CZ silicon single crystal for state classification through the ConvNeXt network. The classification results are shown in Table 2. The confusion matrix is shown in Figure 10.

**Figure 11:**
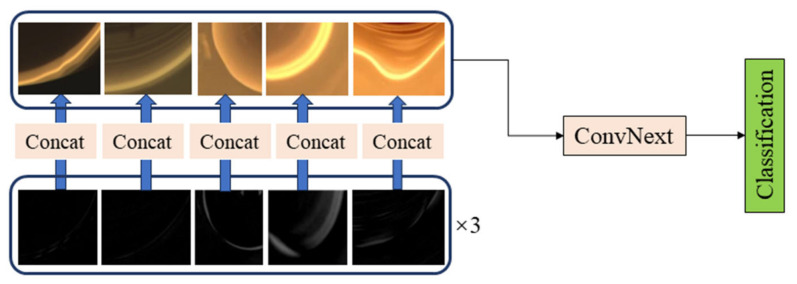
Classification decision of meniscus image and inter-frame difference image fusion.

The experimental results show that the classification accuracy of the meniscus image is 83.3%, while the classification accuracy of the inter-frame difference image is 92.4%. The confusion matrix of the meniscus image classification shows that the image signal has a large error for the classification of normal, loss, and swinging, and has a better classification effect for twisting and squareness. The confusion matrix of the inter-frame difference image fault classification shows that, compared with the meniscus image classification, the detection accuracy of the swinging state has been greatly improved, but the classification of the normal state has decreased. Therefore, the meniscus image and the inter-frame difference image are used for feature fusion to make state classification decisions. The network structure is shown in Figure 11. The classification results are shown in Table 2.

It can be seen from Table 3 that the fusion classification using the meniscus image and the inter-frame difference image achieved a slightly better classification effect than using the inter-frame difference image; the confusion matrix is shown in Figure 9, and it can be seen from the confusion matrix that the classification effect of normal growth and loss using only image signals is poor, while the classification effect of twisting, swinging, and squareness is excellent.

In order to show the superiority of the model, the classification of meniscus data in this study is compared between the ConvNeXt network and the classical excellent classification network. Table 3 shows the classification performance of the above networks for this dataset. Precision, recall, F1-score, and accuracy were also used to evaluate the classification performance. 

It can be seen from Table 3 that the ConvNeXt network is used for feature extraction classification to obtain the best results.

### 4.3. Abnormal State Classification Results by Fusing One-Dimensional Signal and Meniscus Image Signal

After research, this paper found that the detection and classification effect obtained using only one-dimensional signal fusion is better than using only image signals for fusion detection and classification. The detection and classification effect of one-dimensional signals for twisting is better, but the detection and classification effect for loss, normal, swinging, and squareness is poorer, while the detection and classification effect of image signals for swinging, twisting and squareness is better, but the detection and classification effect for normal growth and loss is poorer. From this analysis, only one-dimensional signal fusion for detection and classification or only image signal detection and classification still have their shortcomings, so by making a fusion decision on one-dimensional signals and two-dimensional signals, the effective information of each modality is fully utilized to realize the classification of five states. The network structure used is shown in Figure 10, and the classification results obtained are shown in Table 4.

The confusion matrix in the lower right corner of Figure 10 shows that by using one-dimensional signals and image signals as the input, after feature extraction and modal fusion, the problem of single-dimensional signals being insensitive to some abnormal conditions can be overcome, and the best classification results can be achieved. Table 4 shows that the classification accuracy is as high as 99.2%.

In a later experiment, we compared the performance of the method proposed in this study with other recent silicon single-crystal anomaly detection classification methods, particularly in the aspect of loss detection. To ensure fairness, we used the same dataset and evaluation metrics. Since current research primarily focuses on loss detection, we compared our proposed method with other methods specialized in this task. Existing methods, such as those proposed by Jun Zhang et al. and S Yuting et al., rely solely on image-based loss detection, which neglects the importance of sensor measurement data during the silicon single crystal-growth process [7,8]. This limitation leads to inaccurate anomaly detection. In contrast, our study employs a multimodal fusion technique that integrates both image data and sensor signals, resulting in more reliable classification results. Therefore, we conducted a comparative experiment between our method and the MMFN (Multimodal Fusion Network) method proposed by Lei Jiang et al. [9]. The comparison results are shown in Table 5.

Table 5 indicates that our method outperforms across four evaluation metrics. This improvement is primarily attributed to the use of a more efficient network structure for feature extraction and the introduction of inter-frame difference images, which provide richer modality information. In addition, this study also makes a more detailed classification of the abnormal state of silicon single-crystal growth, including four cases of loss, twisting, swinging, and squareness.

## 5. Conclusions

In this paper, a novel multimodal fusion classification network is proposed for silicon single-crystals’ abnormal growth state classification. The proposed network could leverage multiple data sources, including one-dimensional signals (diameter, temperature, and pulling speed) and video data (meniscus images and frame difference images) to fully monitor the working condition of a single-crystal growth system. First, a novel Dense-ECA-SwinTransformer was proposed to extract temporal-frequency variations from one-dimensional signals’ wavelet transforms. Next, ConvNeXt was utilized to obtain the video abnormal features from meniscus video signals. Finally, we designed a novel multimodal fusion network to fusion the extracted abnormal features from one-dimensional signals and video signals and output the final abnormal detection results. The extensive computational experiments demonstrated that the proposed multimodal fusion classification network could achieve the best classification experimental results with a classification accuracy of 99.2%.

## Figures and Tables

**Table 1:** Classification results of one-dimensional signals in each dimension.

Evaluation Indicators	Precision	Recall	F1-Score	Accuracy
Diameter	0.943	0.947	0.944	0.947
Temperature	0.692	0.701	0.688	0.691
Pulling speed	0.826	0.827	0.826	0.820
One-dimensional signal fusion	0.966	0.969	0.967	0.968

**Table 2:** Meniscus image classification results.

Evaluation Indicators	Precision	Recall	F1-Score	Accuracy
Meniscus image	0.866	0.845	0.850	0.833
Difference image	0.931	0.929	0.930	0.924
Fusion of meniscus image and difference image	0.950	0.947	0.948	0.942

**Table 3:** Classification results of meniscus images.

Classification Network Models	Precision	Recall	F1-Score	Accuracy
VGG	0.849	0.790	0.789	0.780
ResNet	0.832	0.828	0.826	0.820
DenseNet	0.831	0.832	0.829	0.825
MobileNet	0.886	0.821	0.820	0.814
Swin Transformer	0.844	0.838	0.836	0.828
ConvNext (In Figure 6a)	0.866	0.845	0.850	0.833

**Table 4:** Fusion signal classification results.

Evaluation Indicators	Precision	Recall	F1-Score	Accuracy
Fusion of all data	0.992	0.992	0.992	0.992

**Table 5:** Comparison of classification results to existing methods.

Methods	Precision	Recall	F1-Score	Accuracy
**MMFN**	0.993	0.978	0.985	0.986
**Novel multimodal fusion network**	0.996	0.989	0.993	0.993

## Data Availability

The data presented in this study are available on request from the corresponding author. The data are not publicly available due to legal and privacy considerations.
